# Surgical Outcomes of Clitoroplasty in Children with Congenital Adrenal Hyperplasia and Clitoral Hypertrophy: A 19-Year Experience of a Single Surgeon

**DOI:** 10.3390/ijerph182111152

**Published:** 2021-10-23

**Authors:** Jaebeom Jun, Sang Hoon Song, Sungchan Park, Jae Hyeon Han, Kun Suk Kim

**Affiliations:** 1 Department of Urology, Asan Medical Center, University of Ulsan College of Medicine, Seoul 05535, Korea; arhanaz@gmail.com (J.J.); kskim2@amc.seoul.kr (K.S.K.); 2 Department of Urology, Ulsan University Hospital, University of Ulsan College of Medicine, Ulsan 44033, Korea; scpark@amc.seoul.kr; 3 Department of Urology, Korea University Ansan Hospital, Korea University College of Medicine, Ansan 15355, Korea; jfeelgood80@gmail.com

**Keywords:** congenital adrenal hyperplasia, disorders of sex development, feminizing surgery, 21-hydroxylase deficiency

## Abstract

This study aimed to describe the experience with clitoroplasty for clitoral hypertrophy in patients with congenital adrenal hyperplasia of a single surgeon. The medical records of female pediatric patients with congenital adrenal hyperplasia who underwent clitoroplasty at a tertiary referral hospital between 2002 and 2020 were retrospectively analyzed. Three different surgical techniques were applied for clitoroplasty: recession without reduction, reduction and recession, and girth reduction and recession. A total of 104 patients underwent clitoroplasty for clitoral hypertrophy. The median patient age at the time of surgery was 10 months (range, 4 months to 10 years). The operation time was longer in reduction clitoroplasty than in recession clitoroplasty without reduction (median, 153 vs. 111 min, *p* = 0.003). The mean postoperative pain score of the patients did not differ among the different clitoroplasty techniques. During the mean follow-up of 37.7 months, nine (8.6%) patients underwent reperformed clitoroplasty. The rate of reperformed operation was significantly higher in patients who underwent reduction clitoroplasty (17.3%) than in those who underwent recession without reduction (2%) or girth reduction and recession (0%) (*p* = 0.031). Early clitoroplasty in patients with congenital adrenal hyperplasia yielded good mid-term surgical outcomes in terms of cosmesis and recurrence rate, with minimal perioperative complications.

## 1. Introduction

Congenital adrenal hyperplasia (CAH) is an enzymatic defect that results in multiple hormonal imbalances. CAH is one of the most common inherited disorders, with an overall incidence of 1 in 10,000 to 1 in 20,000 live births [1]. The primary goal of treatment in patients with CAH is to replace the insufficient adrenal hormones to maintain normal plasma volume and physiological balance. From a urologic perspective, genital reconstructive surgery is often necessary to restore the normal morphologic and functional anatomy. Mutations in *CYP21A2* result in decreased or lacking 21-hydroxylase activity, which is required for steroidogenesis in the adrenal glands. CAH can cause masculinized genitalia due to excess androgen production. This genital virilization often manifests as clitoral hypertrophy, which ranges from a remarkably enlarged clitoris to a penis-like clitoris in female patients. Most parents of children with clitoral hypertrophy are easily emotionally affected by the appearance of their child’s genitalia [2]. Surgical management can help these patients by providing a normal clitoral appearance, supporting the development of a gender identity consistent with the sex assigned at birth, and enabling appropriate sexual functioning [3]. Additionally, cosmetic clitoral reconstruction in younger patients is generally accepted to relieve parental distress and improve the attachment between parents and children [4,5].

Previous guidelines on the management of CAH support the role of clitoral surgery in severely virilized patients (Prader III–V) when performed by an experienced surgeon using techniques to preserve the erectile function and innervation of the clitoris [3,6]. Recently, the European Society for Pediatric Urology stated that the treatment of children with disorders of sex development, including CAH, is best organized in a multidisciplinary setting, with the patient and the patient’s family playing a central role [7,8]. However, the available scientific information that can help parents understand the optimal surgical procedure, the best timing of clitoral surgery, and the long-term outcomes remain scarce. To allow a family centered multidisciplinary approach and better ensure the physical and emotional growth and development of patients with clitoral hypertrophy and CAH, more data on the outcomes of clitoroplasty are required. Therefore, this study aimed to describe the experience of a single surgeon with clitoroplasty for CAH and clitoral hypertrophy and the surgical outcomes of various clitoroplasty techniques.

## 2. Materials and Methods

After obtaining approval from the institutional review board (Approval No. 2021-0985), we retrospectively reviewed the medical records of all patients with CAH who underwent clitoroplasty performed by a pediatric urologist (K.S.K.) between March 2002 and December 2020 at Asan Medical Center, Seoul, Korea. Perioperative data, including demographic information and variables, such as type of genetic mutation, type of urogenital sinus anomaly, surgical technique, operation time, postoperative pain score, complications, cosmetic outcome, hospital stay duration, follow-up duration, and revision rate, were collected. All patients were assessed by a multidisciplinary team including pediatric urologists, pediatric endocrinologists, gynecologists, geneticists, and pediatric psychiatrists. All surgeries followed the routine preoperative protocol, and the operative details were obtained from the electronic medical records of the patients.

### 2.1. Surgical Techniques

**Figure 1:**
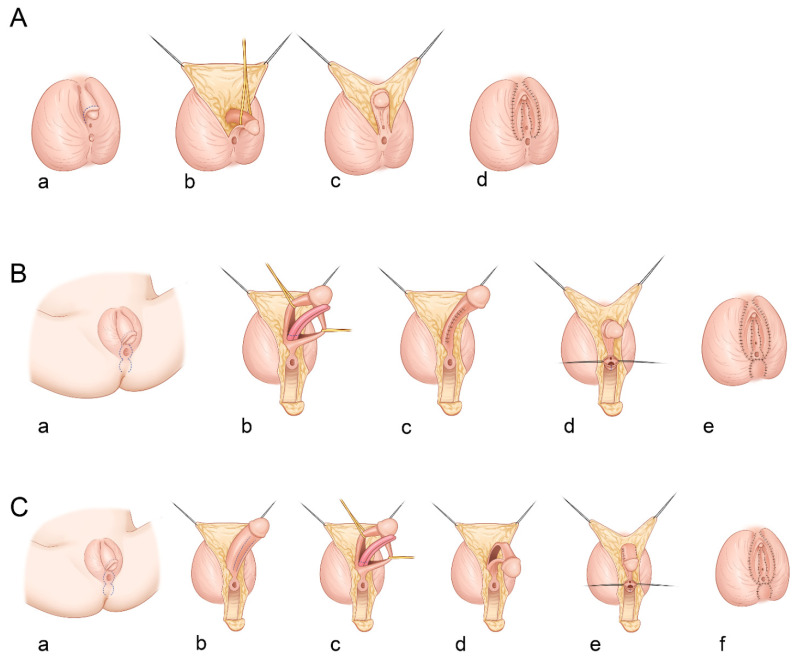
Surgical techniques of clitoroplasty: (**A**) recession clitoroplasty without reduction of corporal tissues (**a**–**d**); (**B**) reduction clitoroplasty method in which the incision line is depicted in blue dot (**a**), the corporal bodies are excised with preservation of the glans clitoris and neurovascular bundles (**b**,**c**), and the posterior vaginal wall is reconstructed with the perineal skin flap (**d**,**e**); (**C**) girth-reduction clitoroplasty in which the glans and corporal bodies are divided in the coronal plane and the ventral portions of the shaft, and glans are excised (**b**,**c**), the dorsal neurovascular bundles are preserved (**d**), and the remaining portion of the clitoris is bent in a hairpin configuration by suturing the exposed surfaces of the corporal bodies (**e**,**f**).

All patients underwent clitoroplasty as a surgical correction of virilization due to CAH. To correct urogenital sinus anomaly, vaginoplasty with a perineal skin flap, urogenital sinus mobilization, or Passerini–Glazel genitoplasty was performed. Three different surgical techniques were used for clitoroplasty. When the clitoral hypertrophy was mild (clitoral size, <2 cm), the clitoris was recessed while preserving all corporal components under the pubis by securing sutures between the dorsal corporal facia near the glans and the inferior margin of the symphysis, as described by Randolph and Hung [9] (Figure 1A). In cases with moderate clitoral hypertrophy, two types of reduction clitoroplasty were performed. One method of reduction clitoroplasty involved excising the corporal bodies with preservation of the glans clitoris and neurovascular bundles, as described by Rajfer et al. [10] (Figure 1B). This nerve-sparing clitoroplasty was achieved through a ventral approach to the corporal bodies and subcoronal circumferential incision proximal to the glans clitoris. Dissection of the corporal bodies continued up to the level of the bifurcation of the crura. The shaft of the clitoris was dissected from the remaining tunica albuginea and neurovascular bundles, and the preserved glans were recessed inferior to the pubic arch. Another method of reduction clitoroplasty was girth-reduction clitoroplasty, which was devised by Robert Fowler and popularized by Hutson et al. [11] (Figure 1C). After degloving the clitoris, the glans and corporal bodies were divided in the coronal plane and the ventral portions of the shaft and glans were excised, preserving the dorsal neurovascular bundles. Thereafter, the remaining portion of the clitoris was bent in a hairpin configuration by suturing the exposed surfaces of the corporal bodies.

### 2.2. Data Collection and Statistical Analyses

The primary outcomes were complications and cosmetic outcomes. Complications were assessed according to the Clavien–Dindo classification. Pain was evaluated according to the Face, Legs, Activity, Cry, Consolability (FLACC) scale [12]. The paired *t*-test was used to compare the FLACC scores between the operation day and the first postoperative day. The total number of opioid analgesics used was counted. The cosmetic outcomes were evaluated by the parents and the surgeon at the follow-up visits. Revision clitoroplasty was indicated when the parents were not satisfied with the cosmetic outcome or when the clitoris measured >2 cm after the surgery. All statistical analyses were performed using SPSS (version 25; IBM Corp, Armonk, NY, USA). Continuous variables have been summarized as medians with interquartile ranges and compared using the Wilcoxon rank test, whereas categorical variables have been summarized as counts and percentages and compared using Fisher’s exact test. A two-sided *p*-value of <0.05 was considered statistically significant.

## 3. Results

The age of the patients at the time of surgery was between 4 months and 10 years (mean, 21 months; median, 10 months) (Table 1). The majority (80%) of the patients underwent clitoroplasty at age <24 months. Most of the patients (96 of 104, 92%) had a genetically confirmed diagnosis of CAH due to 21-hydroxylase (*CYP21A2*) deficiency in 94 patients (90%), 11-β-hydroxylase (*CYP11B1*) deficiency in 1 patient (1%), and P450 oxidoreductase (*POR*) deficiency in 1 patient (1%). Among the 104 children who underwent clitoroplasty, 91 (87.5%) were diagnosed with the salt-wasting type of CAH. The urethrovaginal confluence was assessed to be low in 89 patients (85.6%), intermediate in 3 patients (2.9%), and high in 12 patients (11.5%). Vaginoplasty was performed simultaneously with clitoroplasty in 96 patients (92%), using the Fortunoff flap technique in 92 patients (88.5%) and urogenital sinus mobilization in 4 patients (3.8%).

The patient characteristics according to surgical techniques are summarized in Table 2. The operation time was significantly longer in the reduction clitoroplasty technique (median, 153 min) than in the recession clitoroplasty without reduction technique (median, 111 min) (post hoc analysis, Bonferroni, *p* = 0.003). The hospital stay was also significantly longer in the reduction clitoroplasty technique (median, 7 days) than in the recession clitoroplasty without reduction technique or the girth-reduction-and-recession clitoroplasty technique (median, 6 days each; post hoc analysis, Bonferroni, *p* = 0.005, *p* = 0.040).

Complications greater than Clavien–Dindo grade II were not observed (Table 3). Postoperative bleeding in seven patients (6.7%) was the only perioperative complication identified during the hospital stay, which was conservatively and successfully managed with compressive dressing on the genital area without requiring transfusion. The mean FLACC pain score significantly decreased from 3.8 on the operation day to 2.2 on the first postoperative day (*p* < 0.001). The mean FLACC scores on the operation day and the first postoperative day did not differ among the different clitoroplasty techniques. During the mean follow-up of 37.7 months (median 22.9 months, range 1.0–179.7), the parents of two patients (1.9%) were dissatisfied with the postoperative cosmetic outcome. Clitoral hypertrophy recurrence was observed in 11 patients (10.5%), 9 (8.6%) of whom underwent reperformed clitoroplasty. The reperformed operation rate was significantly higher in the reduction clitoroplasty technique (17.3%) than in the recession-without-reduction (2%) or girth-reduction-and-recession (0%) techniques (*p* = 0.031).

## 4. Discussion

This study shows the satisfactory mid-term surgical outcomes of early clitoroplasty in patients with CAH. The experience at our tertiary referral hospital showed an overall high surgical success rate, with minimal immediate surgical complications in patients managed by a multidisciplinary team consisting of pediatric urologists, endocrinologists, gynecologists, geneticists, and pediatric psychiatrists. The results of this study add to the clinical information that can help the decision making of both the physicians and parents of children with clitoral hypertrophy who are candidates for surgical correction.

The timing of clitoroplasty with or without vaginoplasty in our study was “early”, as the median age of the patients at surgery was 10 months. The value of early corrective surgery for CAH has been supported by previous studies. Performing feminizing genitoplasty before 2 years of age and offering single-stage interventions were recommended by the Fourth World Congress of the International Society of Hypospadias and Disorders of Sex Development Surgery and the American Academy of Pediatrics [13,14,15]. Sturm et al. reported that the median age of patients who underwent combined clitoroplasty or vaginoplasty was 11.3 months at academic medical centers in the United States [16]. Wisniewski et al. reported that the optimal timing for genital reconstruction in most patients with CAH is during infancy and early childhood [17]. For some patients, delaying corrective surgery until adolescence, with the anticipation of undergoing genital surgery, may add to the stigma of genital atypicality [18]. Successful early surgical correction can reduce the stigma associated with genital atypicality [19]. Our results support the value of early clitoroplasty by demonstrating the high satisfaction of the patients’ parents with the cosmetic outcome and the low recurrence rate of clitoral hypertrophy.

There is an ongoing debate about the optimal timing for genital surgery in patients with disorders of sex development (DSD) [2]. CAH is the most common diagnosis among the 46, XX DSD group [3]. Some proponents for late surgery favor this approach to involve the patient in the decision process of genital surgery [20]. Lean et al. reported that cosmetic results do not differ between early and late surgery [21] Burgu et al. stated that patients who underwent flap vaginoplasties prepubertally experienced more complications than those who received this surgery postpubertally [22]. However, pubertal or postpubertal genital surgery bears a much greater risk of surgical morbidity, compared with early surgery in childhood, because blood loss and infection are more common in adult genital surgery [20]. Evidence on whether early or late surgery is better for patients with CAH is still lacking [2,20].

No consensus has been reached on the best technique for reducing the clitoral volume that can provide satisfactory long-term cosmetic or functional outcomes. Even the decision to perform genital surgery in children with clitoral hypertrophy is controversial [23]. Recently, European countries strongly condemned sex-normalizing treatments and medically unnecessary surgery in the genitals of infants with disorders of sex development [24]. It is recommended that surgery should be performed only by experienced surgeons/urologists after extensive discussions about the risks and benefits of surgical treatment [25]. However, it is not uncommon for clinicians to fail to discourage the parents of children with clitoral hypertrophy due to CAH from choosing surgical correction of their child’s atypical genital anatomy [19]. All parents of the patients included in this study voluntarily visited our urology clinic and provided consent for their child to undergo clitoroplasty, mostly simultaneously with vaginoplasty, owing to the social and psychological impact of the child’s genital appearance.

Various surgical techniques for the management of clitoral hypertrophy have been reported in the literature. When clitoroplasty was first introduced, clitoridectomy was recommended on the basis of the theory that the clitoris has no function [26]. However, after the clitoris was discovered to play an important role in sexual sensitivity, techniques for preserving the clitoris and neuromuscular bundles began to be suggested [10,27]. Rajfer et al. described excision of the corporal bodies with preservation of the glans clitoris and neurovascular bundles [10]. By incising the ventral skin of the clitoris, the dorsal aspect of the clitoris and the neurovascular bundles are left untouched during the partial amputation of the corporal bodies. Poppas et al. popularized the technique of nerve-sparing ventral clitoroplasty, in which Buck’s fascia is preserved during the degloving of the clitoris and the dissection of the corporal bodies [28]. Kogan et al. demonstrated subtunical reduction of the cavernosal tissue with preservation of the entire tunica albuginea to completely save the neurovascular bundles [29]. Hutson et al. reported girth-reduction clitoroplasty in which the glans and corporal bodies are divided in the coronal plane, and the ventral side is excised from the corpora while protecting the neurovascular bundles [11]. Randolph and Hung described a recession clitoroplasty technique that preserves all corporal components and secures the clitoris under the margin of the symphysis [9]. Pippi Salle et al. developed corporal-sparing dismembered clitoroplasty in which each hemicorpora is placed in a dartos pouch of each labia majora [30].

The perioperative complication rate in this study was low regardless of the surgical technique applied. This concurs with previous reports on the outcome of feminizing genitoplasty for CAH. In a study using large-scale data analysis, Roth et al. showed a 4.0% perioperative surgical complication rate in a cohort of 544 patients with CAH who underwent female genital restoration surgery at a median age of 10 months [31]. In their study, 2.0% of the patients underwent reoperation before discharge. A 13.8% readmission rate for any reason within 30 days was reported. In the present study, reperformed clitoroplasty was needed in nine patients (8.7%). The reperformed clitoroplasty rate was significantly lower in the girth-reduction clitoroplasty described by Hutson et al. (0%) than in the reduction clitoroplasty described by Rajfer et al. (17.3%) during a similar median follow-up duration (26.1 vs. 23.5 months). This difference is notable considering the similar age at surgery, opioid use, and hospital stay among patients who underwent the two reduction clitoroplasty techniques. Given the older age and higher incidence of a high urethrovaginal confluence type in patients who underwent reduction clitoroplasty, despite the lack of statistical significance, a selection bias may exist related to the tendency of the surgeon to select a reduction technique in more virilized or severe clitoral hypertrophy cases.

It was reported that the majority of CAH patients undergo simultaneous vaginoplasty during their initial female genital restoration surgery [14,31]. Roth et al. demonstrated that 92% of patients with CAH underwent a vaginal procedure from a survey in the United States [31]. Yankovic et al. reported that 75% of specialist surgeons who practice feminizing surgery for CAH and replied in a survey perform a combined procedure of clitoroplasty, labiaplasty, and vaginoplasty [14]. In the present study, vaginoplasty was performed simultaneously with clitoroplasty in 92.3% of patients. The majority of our patients (88.4%) showed low vaginal confluence. Therefore, less complicated surgical techniques such as Fortunoff flap or simple cutback were useful and sufficient to separate urethral and vaginal opening and to bring them down to the vulva.

First, the retrospective design and possibility of selection bias in terms of the surgical techniques could be limitations of our study. Second, we did not use an objective scoring system to measure the parents’ satisfaction regarding the cosmetic results. Therefore, the evaluation of cosmetic outcomes by parents was subjective. In this study, patients with a Prader scale of 0 or 1 after surgery without any cosmetics-related complaint from the patient or patient’s parents were regarded as a good cosmetic outcome. Third, data on the long-term follow-up of the sexual and emotional functions of the patients were not collected in this study. Therefore, a long-term follow-up and postpubertal evaluation of the sexual function, as well as the satisfaction of patients with the surgical outcomes, are warranted.

## 5. Conclusions

Early clitoroplasty in patients with CAH showed successful surgical outcomes during a mid-term follow-up duration in terms of cosmesis and recurrence rate, with a low perioperative complication rate. Studies with a long-term follow-up and postpubertal evaluation of the sexual function and satisfaction of patients with the surgical outcomes are warranted.

## Figures and Tables

**Table 1:** Baseline demographic and clinical characteristics of patients.

Demographic/Clinical Variables	N = 104
	*n* (Range or %)
Age (months)	10 (4–120)
<2 years	83 (79.8%)
>2 years and before adolescence	21 (20.2%)
Follow-up period (months)	37.7 (0.8–178.9)
CAH type	
Salt-wasting type	91 (87.5%)
Simple virilizing type	10 (9.6%)
Unknown	3 (2.9%)
Confluence type	
Low confluence	89 (85.6%)
High confluence	12 (11.5%)
Intermediate confluence	3 (2.9%)

**Table 2:** Surgical details of the clitoroplasty techniques.

	Recession without Reduction (*n* = 46)	Reduction and Recession (*n* = 47)	Girth Reduction and Recession (*n* = 11)	*p*-Value
Median age (IQR) (months)	9 (4–120)	13 (4–108)	9 (6–13)	0.051 *
Operation time (min)	111 (54–236)	153 (60–365)	122 (65–175)	0.002 *
Hospital stay (days)	6 (3–11)	7 (5–12)	6 (5–6)	0.001 *
Vaginoplasty technique				0.345 ^‡^
UG mobilization	2	2	0	
Fortunoff flap	29	31	11	
Cutback	11	10	0	
No vaginoplasty	4	4	0	
CAH type				0.79
Salt-wasting type	40	40	11	
Simple virilizing type	4	6	0	
Unknown	2	1	0	
Confluence type				0.067 ^‡^
Low confluence	40	40	9	
Intermediate confluence	1	0	2	
High confluence	5	7	0	

IQR, interquartile range; UG mobilization, urogenital sinus mobilization; CAH, congenital adrenal hyperplasia. * Kruskal–Wallis test. ^‡^ Fisher’s exact test.

**Table 3:** Postoperative complications and surgical outcomes.

	Recession without Reduction (*n* = 46)	Reduction and Recession (*n* = 47)	Girth Reduction and Recession (*n* = 11)	*p*-Value
Immediate complications				0.244
Clavien–Dindo grade I	1	6	0	
Bleeding without transfusion	1	6	0	
FLACC score				
Operation day	3.73 (2.36)	4.14 (2.43)	3.27 (1.42)	0.571
Postoperative day 1	2.09 (1.86)	2.61 (2.44)	1.64 (2.11)	0.337
Opioid use	37.0%	54.3%	8.7%	0.248
Follow-up period (months)	28.7 (1.3–102.0)	48.0 (0.8–179.7)	31.3 (2.0–65.9)	0.057
Long-term cosmetic dissatisfaction of patients	1	1	0	1.000
Clitoral hypertrophy recurrence	2	9	0	0.047
Reperformed surgery	1	8	0	0.031

FLACC, Face, Legs, Activity, Cry, Consolability.
